# A new unfolded pocket mesh for pre-pectoral direct to implant breast reconstruction: first case report

**DOI:** 10.1093/jscr/rjag163

**Published:** 2026-03-20

**Authors:** Stefano Formica, Michele Antonio De Riggi, Gianmarco Polverino, Francesca Russo, Nicola Rocco, Mena Pia Panico, Massimiliano D'aiuto, Francesco D'Andrea

**Affiliations:** Aesthetic Reconstructive and Plastic Surgery, University of Naples Federico II, Naples 80131, Italy; General and Breast Surgery Department, Boscotrecase Hospital, 80042 Azienda Sanitaria Locale Napoli 3 Sud, Italy; Aesthetic Reconstructive and Plastic Surgery, University of Naples Federico II, Naples 80131, Italy; Aesthetic Reconstructive and Plastic Surgery, University of Naples Federico II, Naples 80131, Italy; General Surgery, University of Naples Federico II, Naples 80131, Italy; General and Breast Surgery Department, Boscotrecase Hospital, 80042 Azienda Sanitaria Locale Napoli 3 Sud, Italy; General and Breast Surgery Department, Boscotrecase Hospital, 80042 Azienda Sanitaria Locale Napoli 3 Sud, Italy; Aesthetic Reconstructive and Plastic Surgery, University of Naples Federico II, Naples 80131, Italy

**Keywords:** breast reconstruction, breast cancer, titanium-coated polypropylene mesh

## Abstract

After mastectomy for breast cancer, implant-based breast reconstruction (IBBR) is the main approach to define a new breast shape. While conventionally implants were typically located beneath the pectoralis major muscle, the choice is moving to place directly the implant in the pre-pectoral space covered by subcutaneous tissue, also supported by advancements like acellular dermal matrices and synthetic meshes. We report the first case of a 50 years old patient affected by breast cancer suitable for mastectomy and pre-pectoral IBBR with the use of a new titanium-coated polypropylene mesh (TCPM) TiO2Mesh BRA®. No complications occurred post-operatively. 18-month follow-up, the patient was disease-free with a satisfactory cosmetic outcome. TCPM provides a large pocket for implant support, making it possible to place a high-volume prosthetic implant, and reducing risks of implant dislocation. This new mesh used by our team improves pre-pectoral IBBR outcomes, towards customized breast reconstruction for women.

## Introduction

Breast cancer represents the most common tumor among women [1]. Over the last decades, surgical options for breast cancer treatment have evolved from modified radical mastectomy and standard breast-conserving surgery to a so-called oncoplastic approach in both breast-conserving surgery and mastectomy, with the development of skin- and nipple-sparing mastectomies and several options for immediate reconstruction [2, 3]. Implant-based breast reconstruction (IBBR) represents the most common reconstructive procedure following conservative mastectomies. It can be achieved through a two-stage approach or a single-stage direct-to-implant (DTI) approach. The implant location has progressively shifted from a sub-pectoral to a pre-pectoral position, covered only by subcutaneous tissue. The use of Acellular Dermal Matrix (ADM) and synthetic meshes as titanium-coated polypropylene mesh (TCPM) has also contributed to the further diffusion of pre-pectoral reconstructions [4, 5]. We report the first case of immediate pre-pectoral implant-based breast reconstruction using a new TCPM that wraps the implant.

## Case report

**Figure 1:**
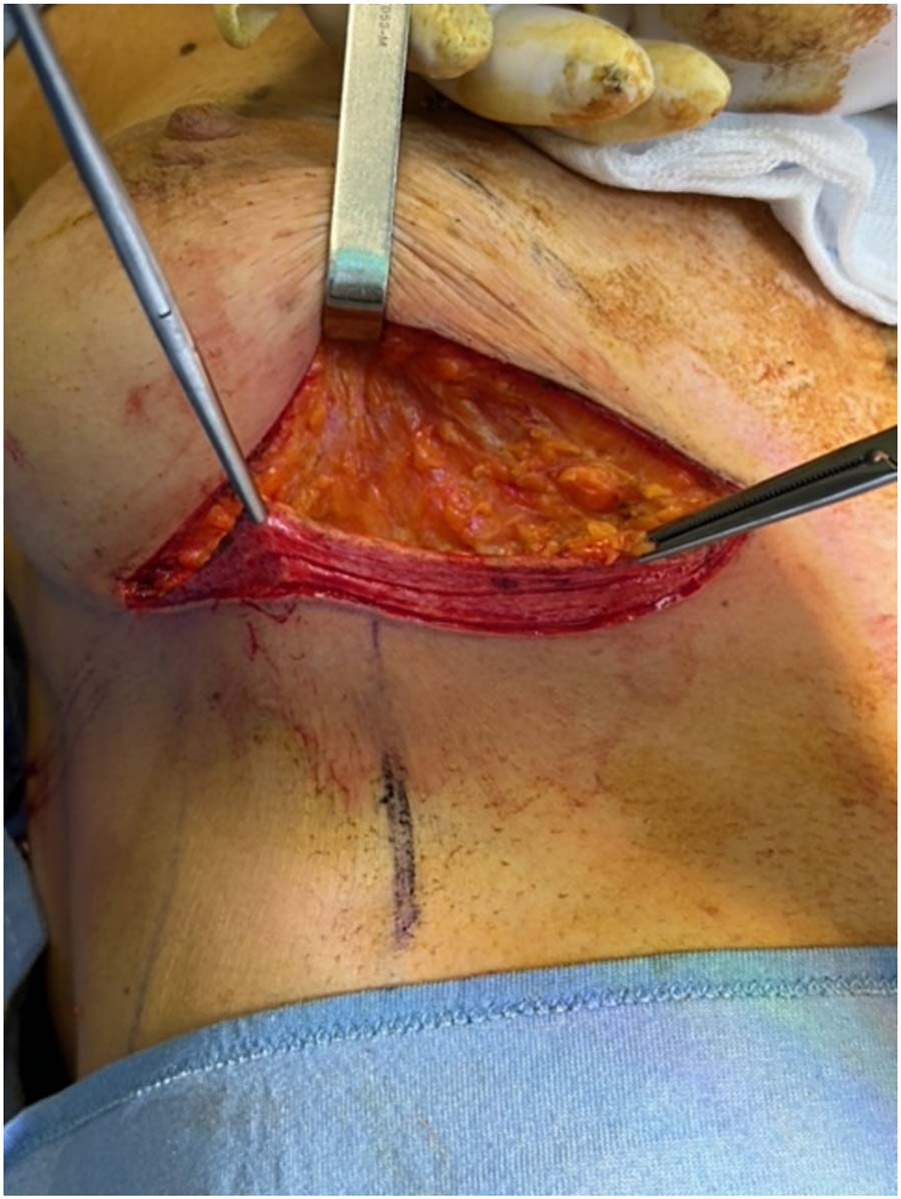
Inframammary skin incision with dermal flap.

**Figure 2:**
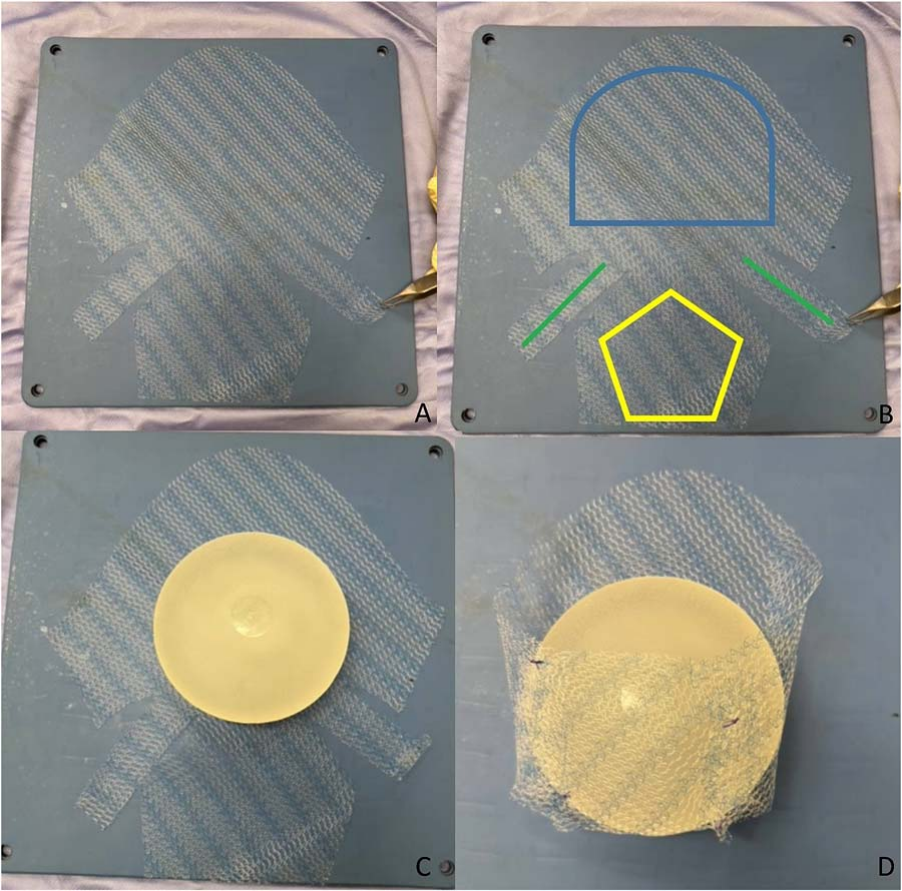
The mesh and creation of pocket around the implant.

**Figure 3:**
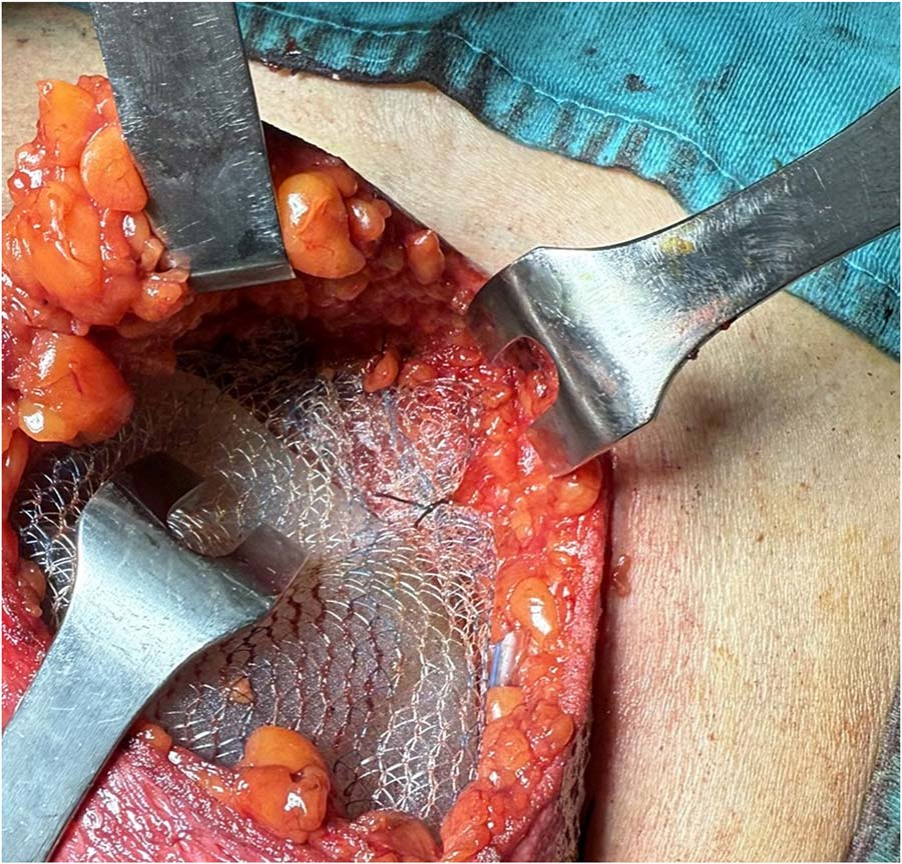
Anchoring the mesh to pectoralis fascia.

**Figure 4:**
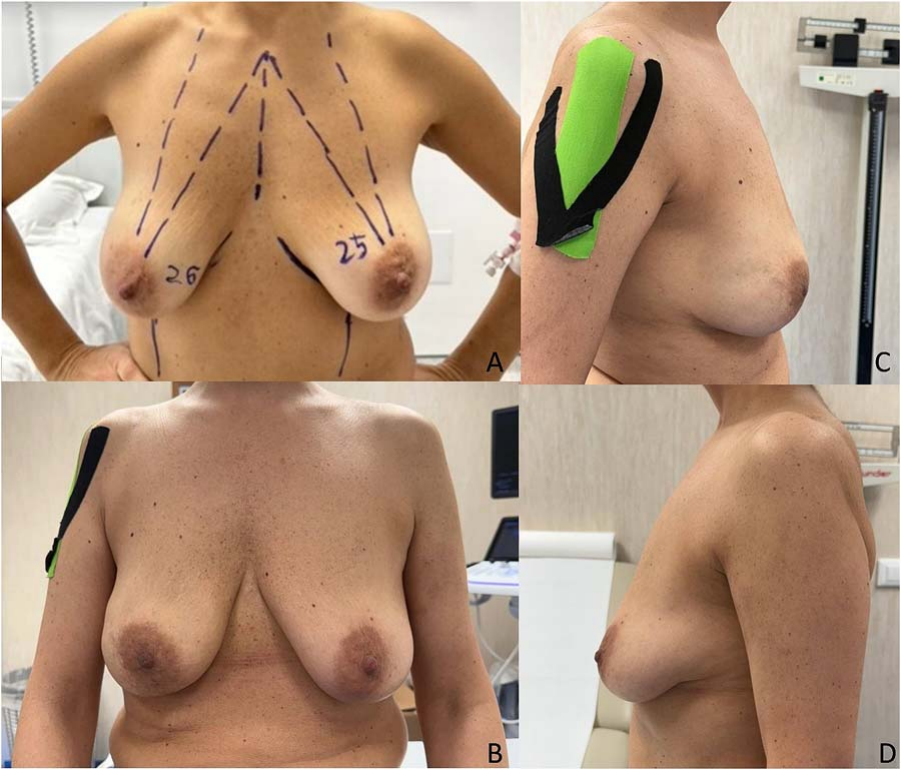
Preoperative photo (A) and postoperative control after 18 months (B–D).

We report the case of a 50-year-old woman affected by right metacentric invasive luminal A breast cancer without any sign of clinical or radiologic axillary node involvement. The Multidisciplinary Board agreed for upfront surgery and the patient was scheduled for a right mastectomy with preservation of nipple areola complex and sentinel lymph node biopsy. The patient presented a grade 3 breast ptosis, large breast volume (Right Sternal Notch to Nipple (SN-N) distance of 26 cm and left SN-N distance of 25 cm) with a superficial tissue coverage over the gland from 1 to 2 cm (type 2 according to Rancati score) [6]. In a shared decision-making process, we opted for an immediate pre-pectoral implant-based breast reconstruction with a 450 cc round silicone gel implant with a moderate projection (Mentor®) and a titanium-coated polypropylene TiO2 mesh BRA® (BioCer Entwicklungs- GmbH, Bayreuth, Germany). This was intended to create a tailored pocket for the breast implant in order to enhance the coverage of the implant, pocket control and potentially reducing complications. The patient refused contralateral breast symmetrization. Written consent was obtained by the patient for the use of implantable medical device (mesh and prosthesis) and relative images. Surgical procedure started with incision along the inframammary fold over a length of about 8 cm (Fig. 1). Conservative mastectomy was performed as oncological procedure, subsequently we evaluated the good thickness of skin mastectomy flaps which was crucial to realize the pre-pectoral reconstruction. Our reconstruction option involved creating a new breast using an implant covered by a TCPM that envelops almost completely the implant. The TiO2Mesh BRA® is a pre-shaped unfolded mesh. The mesh was initially laid out on the sterile operating table. *The implant was placed in the center of the upper part of the mesh, ensuring direct contact between the mesh and the implant’s anterior surface while the lower part and lateral wings were folded over to complete the pocket.* Two reabsorbable 2/0 stitches were placed to approximate the edges of the lateral strings to lower portion (Fig. 2A–D). Then the implant/pocket unit was positioned in the pre-pectoral space and fixed to the pectoralis major muscle fascia with a three re-absorbable 2/0 suture, one placed on the center of the upper edge of the mesh, the second and third on the edges of the upper border medially and laterally (Fig. 3). Skin flaps were sutured, and one Blake drain was placed in subcutaneous site, the other placed in axillary space. Prophylactic antibiotic was given as a one-time dose of 2 g cefazolin. Drainage tubes were extracted when the fluid volume was beneath 30 ml each day for at least two consecutive days. No immediate post-operative complications were reported. At 18 month follow-up the patient did not experience any complications, she is oncologically free of disease, with a pleasant reconstructive result and a high level of satisfaction with her reconstructed breast (Fig. 4A–D). The aesthetic outcome as assessed by surgeons was overall acceptable. Patient’s post-operative quality of life and satisfaction levels was measured with BREAST-Q© Version 2.0 questionnaire [7] and showed good satisfaction with the post-operative outcomes (Table 1).

**Table 1:** EORTC European Organization for Research and Treatment of Cancer; QLQ-C30 quality of life questionnaire core 30; QLQ-BRECON 23, quality of life questionnaire breast reconstruction module.

Questionnaire	
QLQ-C30	
Psychological well-being	1.7
Fatigue symptoms	1.9
Overall quality of life	5.2
QLQ-BRECON23	
Sexual well-being	1.8
Sensation of the operative area	2.1
Satisfaction with the reconstruction	3.4

Low score is an indicator of high quality.

High score is an indicator of high quality of life.

## Discussion

**Figure 5:**
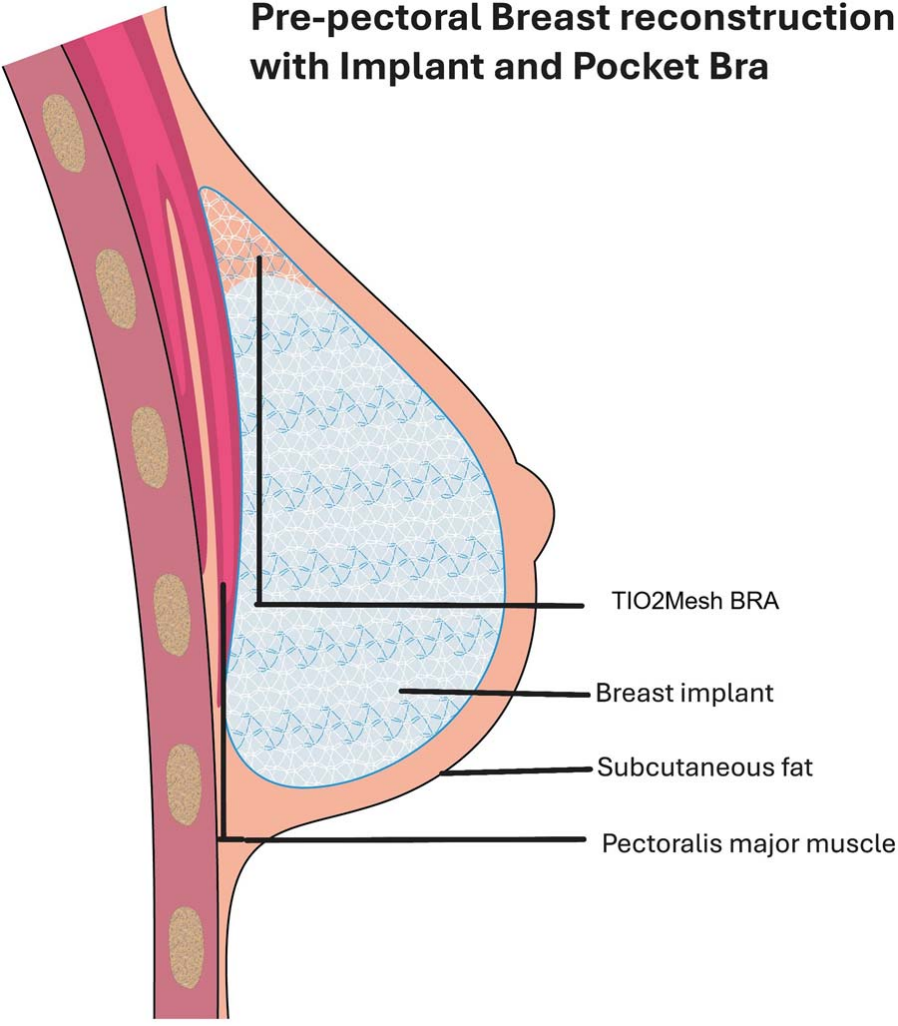
Illustration of position of mesh and implant.

Pre-pectoral IBBR is gaining popularity among breast surgeons, facilitated by the introduction of ADMs and synthetic meshes. The most important factor influencing the successful of pre-pectoral breast reconstruction is sufficient fatty tissue coverage underneath the breast skin [8]. Subcutaneous thickness above 2 cm represents an important indication for good tissue coverage (Rancati Score). Patient with low and moderate coverage (flap thickness <10 mm or between 1 and 2 cm) could benefit from the use of mesh which adds a layer of protection and reducing complication rates such as postal codesular contracture or inflammatory response, improving aesthetic outcomes [9]. The use of TCPM for breast reconstruction was approved in Europe in 2008. Currently, only one commercially available synthetic mesh offers a preformed pocket design that fully encloses the implant [10, 11] Complication rates varies among studies; several series showed seroma, infection, and implant loss similar or lower compared with ADM, influenced by radiotherapy, BMI, and smoking, ranged around 20%–35% [12, 13]. Vidya and Iqbal elaborated a cost analysis comparing meshes with ADM concluding that meshes were cheaper (meshes: €500 vs. ADM £1800–3000); however, the cost of the meshes varies across countries [14]. The high cost and lower availability of ADM make a more practical and economically feasible alternative, with comparable complication rates [15]. TiO2Mesh BRA® is not preshaped and must be manually adapted according to the width of the implant, avoiding any space remaining between the mesh and the implant. It supports the weight of the prosthesis, which lies gently in the pocket, reducing the risk of dislocation and upward migration—complications that are not uncommon in sub-pectoral reconstruction or after radiotherapy (Fig. 5). The use of a tailored titanium-coated polypropylenemesh could be a reliable and cost-effective option to improve the outcomes of implant-based pre-pectoral breast reconstruction, towards a customized reconstruction for the women.
